# ASIC3 knockout alters expression and activity of P2X3 in muscle afferent nerves of rat model of peripheral artery disease

**DOI:** 10.1096/fba.2021-00156

**Published:** 2022-01-25

**Authors:** Lu Qin, Qin Li, Jianhua Li

**Affiliations:** ^1^ Heart and Vascular Institute The Pennsylvania State University College of Medicine Hershey Pennsylvania USA

**Keywords:** ASIC, blood pressure, dorsal root ganglion, hindlimb ischemia, muscle afferent, P2X, peripheral artery disease, sympathetic nervous activity

## Abstract

In peripheral artery disease (PAD), the metaboreceptor and mechanoreceptor in muscle afferent nerves contribute to accentuated sympathetic outflow via a neural reflex termed exercise pressor reflex (EPR). Particularly, lactic acid and adenosine triphosphate (ATP) produced in exercising muscles respectively stimulate acid sensing ion channel subtype 3 (ASIC3) and P2X3 receptors (P2X3) in muscle afferent nerves, inducing the reflex sympathetic and BP responses. Previous studies indicated that those two receptors are spatially close to each other and AISC3 may have a regulatory effect on the function of P2X3. This inspired our investigation on the P2X3‐mediated EPR response following AISC_3_ abolished, which was anticipated to shed light on the future pharmacological and genetic treatment strategy for PAD. Thus, we tested the experimental hypothesis that the pressor response to P2X3 stimulation is greater in PAD rats with 3 days of femoral artery occlusion and the sensitizing effects of P2X3 are attenuated following ASIC_3_ knockout (KO) in PAD. Our data demonstrated that in wild type (WT) rats femoral occlusion exaggerated BP response to activation of P2X3 using α,β‐methylene ATP injected into the arterial blood supply of the hindlimb, meanwhile the western blot analysis suggested upregulation of P2X3 expression in dorsal root ganglion supplying the afferent nerves. Using the whole cell patch‐clamp method, we also showed that P2X3 stimulation enhanced the amplitude of induced currents in muscle afferent neurons of PAD rats. Of note, amplification of the P2X3 evoked‐pressor response and expression and current response of P2X3 was attenuated in ASIC3 KO rats. We concluded that the exaggerated P2X3‐mediated pressor response in PAD rats is blunted by ASIC_3_ KO due to the decreased expression and activities of P2X3 in muscle afferent neurons.

**Abbreviations**
ASIC3: acid sensing ion channel subtype 3
ATP: adenosine triphosphate
BP: blood pressure
DRG: dorsal root ganglion
EPR: exercise pressor reflex
KO: knockout
MAP: mean arterial pressure
P2X3: purinergic receptor subtype 2 unit X3
PAD: peripheral artery disease
SNA: sympathetic nerve activity

## 1 INTRODUCTION

The sympathetic nervous system is activated during exercise.^1^ This leads to increases in arterial blood pressure (BP), heart rate (HR), myocardial contractility, and vascular tone in inactive beds.^2^, ^3^ Two basic mechanisms contribute to these exercise responses. The first, termed ‘central command,’ suggests that motor and sympathetic nervous activations occur in parallel.^4^ The second, termed the ‘exercise pressor reflex (EPR)’, suggests that afferents in contracting skeletal muscle are engaged and an autonomic reflex is initiated.^5^, ^6^ This system responds to metabolic stimulation (i.e., ‘metaboreceptor’ stimulation) as well as to mechanical deformation of the muscle afferent receptive field (i.e., ‘mechanoreceptor’ stimulation). Group IV afferents are thought to be predominantly metabosensitive afferents, and group III afferents are thought to be predominantly mechanically sensitive.^7^ When these receptors are stimulated, thin‐fiber muscle afferent nerves are engaged, cardiovascular nuclei in the brain stem are activated, sympathetic nervous activity (SNA) increases, and BP rises. Considering the important role of EPR in the sympathetic and BP regulation during exercise, there has been a great attention on the abnormalities in the EPR in a various cardiovascular disease, for example, peripheral artery disease (PAD).

PAD is an atherosclerotic disease with a decrease in blood flow to the arteries of the lower extremities. However, the autonomic adjustments to exercise in PAD are poorly understood. It has been observed that a greater increase in arterial BP, which is attributed to the exaggerated exercise pressor reflex, in PAD patients than in normal subjects during walking.^8^ Subsequent studies using a rat model of PAD induced by femoral artery occlusion have suggested that SNA and BP responses to activation of muscle metabolite‐sensitive and mechano‐sensitive afferents contributing to the exercise pressor reflex are increased to a greater degree in PAD than in controls.^9^, ^10^ Chemically sensitive ASICs and P2Xs are preferentially presented on group III and IV sensory nerves (neurons) in skeletal muscle and involve the functional roles played by lactic acid and adenosine triphosphate (ATP), respectively.^11^ When lactic acid and acidic phosphate are injected into the hindlimb circulation, they stimulate thin fiber muscle afferent nerves via ASIC_3_ and evoke a muscle metaboreflex in both rats whose hindlimb is freely perfused and rats whose femoral artery is occluded. In previous studies,^12^, ^13^ the activities of the two key families of metabolite receptors: acid sensing ion channels and purinergic receptors P2X (P2XRs), are augmented in both simulated rat PAD model. Of note, the effects of those lactic metabolites appear to be greater in occluded rats.^13^ In occluded rats, a greater reflex response is observed after intra‐arterial administration of α,β‐methylene ATP (αβ‐me ATP), an activator of P2X_3_, into the hindlimb muscles.^14^ This partly addressed the underlying mechanism for the exaggerated BP response in the PAD patients.

More interestingly, the ASICs and P2XRs may interact with each other while they regulate the BP and other sensory responses. A recent study^15^ demonstrated a synergetic effect of acid and ATP in cultured dorsal root ganglion (DRG) neurons in vitro, as well as in the process of pain behavior during the ischemic situations. Specifically, it was revealed that the ASIC3 and P2X3 are not only co‐expressed in the rat DRG neuron, but also that there is a unidirectional regulatory effect of the ASIC3 activation on the P2X_3_ during development of the P2X currents. It was suggested that the ASIC3 and P2X3 are spatially close with each other and possibly form a cognate receptor in regulating the activities of sensory neurons.

Results from the above studies inspired us to explore the EPR response mediated by the P2X3 when the ASIC3 is replenished. This is likely to bring a novel foundation for the future pharmacological and genetic therapy on the PAD patients. Therefore, in the present study, we determined whether the ASIC_3_ has a mediating effect on the activity of ATP‐P2X3 pathway in PAD rats with femoral artery occlusion. Thus, we postulated that inhibition of ASIC_3_ pathway would alter reflex P2X‐mediated BP response in PAD rats. The model of ASIC3 knockout (KO) provides a useful tool to study this important issue in PAD rats. We specifically postulated in this report that amplified BP response to αβ‐me ATP injected the hindlimb circulation in PAD rats would be attenuated following ASIC_3_ KO via altering the activity of ATP‐P2X3 signaling pathway. Our overall hypothesis was that femoral artery occlusion increases the ATP‐P2X3 activity, which was verified by expression of P2X3 in DRG, responses of P2X currents in muscle DRG neurons and BP response to αβ‐me ATP, in wild type (WT) rats; whereas the effects of femoral artery occlusion are blunted in ASIC3 KO rats.

## 2 METHODS

### 2.1 Ethical approval

The animal experimental procedures were performed in compliance with the National Institutes of Health guidelines and were approved by *the Institutional Animal Care and Use Committee* of the Pennsylvania State College of Medicine. Previous study results observed in either humans or in animals suggests sex difference in the EPR response.^16^, ^17^ It has been also indicated that there is a regulatory role of estrogen hormone on the expression and function of P2X3.^18^ Nonetheless, female animals were firstly investigated within the scope of the present study although additional studies are needed to study sex difference. Female Wistar Kyoto (WKY) rats and female ASIC_3_ KO rats (200–300 g) were obtained from Charles River Laboratory and housed in individual cages with free access to food and water and they were kept in a temperature‐controlled room (25°C) on a 12‐h/12 h light/dark cycle.

### 2.2 Experimental animals and categorization

**FIGURE 1:**
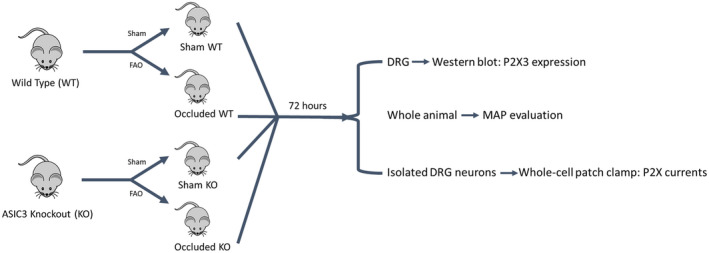
A graphical diagram of the study design. DRG, dorsal root ganglion; FAO, femoral artery occlusion; MAP, mean arterial pressure

WKY transgenic ASIC_3_ KO rats and WKY WT rats were involved in the present study. The ASIC_3_ KO strain rats were breed, obtained, and validated by the same resource with the previous studies.^19^, ^20^ Genotyping was utilized to validate the KO. Either ligation or sham surgery procedures were performed. Those that underwent the femoral artery occlusion served as ‘Occluded WT rats’ and ‘Occluded KO rats’, whereas those underwent sham surgeries served as ‘WT rats’ and ‘KO rats’. Thus, in the present study four groups of animals were included: WT, Occluded WT, KO, and Occluded KO. A graphical diagram of the study design is shown in Figure 1.

### 2.3 Femoral artery occlusion

After being anaesthetized with an isoflurane‐oxygen mixture (2%–5% isoflurane in 100% oxygen), femoral artery ligation was performed as previously described.^21^, ^22^ In brief, an ~1 cm surgical incision was made on the skin on the groin. After carefully cutting the fascia and removing the soft tissue around the veins, the femoral artery was exposed, dissected, and ligated with a surgical suture ~3 mm distal to the inguinal ligament. For the sham surgery, the same procedures were performed except that a suture was placed below the femoral artery without ligating the artery. Buprenorphine hydrochloride (0.05 mg/kg, subcutaneously) was administered prior to the surgery for postoperative pain relief. Following the surgery, the animals were kept in the surgery room for 2–3 h for observation, and then returned to the animal facility for 3 days before experiments were performed.

### 2.4 Western blot analysis

Western blotting was performed to examine the protein expression of the ASIC3 and P2X3 receptor in the L4‐6 DRG tissues of both control limbs and occluded limbs. The rats were anesthetized with an isoflurane‐oxygen mixture and then euthanized by cervical dislocation. After removal of the spinal cord, the L4‐6 DRGs in both sides were dissected and the tissue samples were homogenized with ice‐cold radioimmunoprecipitation assay (RIPA) buffer with proteinase inhibitor cocktail (Sigma‐Aldrich). The samples were then centrifuged at 4°C with 13,000 rpm for 25 min. The supernatants were carefully collected, and the total protein concentration was determined with a bicinchoninic acid (BCA) assay kit (Pierce Biotechnology). The tissue extraction was then divided into the tubes and stored at −80°C until analysis.

Then, the supernatant samples with 30 μg of protein were boiled at ~95°C for 5 min in SDS sample buffer and then loaded onto 10% Mini‐Protean TGX Precast gels (Bio‐Rad Lab) for gel electrophoresis. Following electrophoresis, the proteins were electrically transferred to a polyvinylidene fluoride (PVDF) membrane. The membrane was then blocked with 5% nonfat milk in 0.1% Tween‐TBS buffer (TBST) for 1 h and incubated with a mouse anti‐ASIC3 (1:400, Abcam) and anti‐P2X3 (1:500, Santa Cruz) primary antibodies overnight at 4°C.

After overnight incubation with the primary antibody, the membrane was incubated with horseradish peroxidase‐conjugated anti‐rabbit and anti‐mouse secondary antibody (1:1000, Abcam) at room temperature for 1 h, and the immunoreactivity was visualized using an enhanced chemiluminescence system (Cell Signaling Tech). The membrane was then stripped and incubated with an anti‐β‐actin antibody. The densities of the P2X3, ASIC3, and β‐actin bands were examined using the NIH Scion Image Software.

### 2.5 Electrophysiology

Rats were anesthetized with an isoflurane‐oxygen mixture (2%–5% isoflurane in 100% oxygen). The skin was incised and pulled away from the underlying muscle tissues, and the fluorescent retrograde tracer DiI (60 mg/ml) was injected into the white portion of the gastrocnemius muscle to label muscle DRG neurons. An injection volume of 1 µl was administered, and the injection was repeated three times at different locations.^12^ The injection needle was left in the muscle for 5–10 min to prevent leakage of the tracer. The skin overlying the muscle was then sutured. The animals were returned to their cages for 4–5 days to allow the retrograde tracer to be transported to DRG neurons.

To perform the patch‐clamp experiments, the rats were anesthetized with an isoflurane‐oxygen mixture and then euthanized by cervical dislocation. The L4‐6 DRGs were quickly removed and transferred immediately into Dulbecco's modified Eagle's Medium (DMEM). The DRGs were minced, and the ganglion fragments were processed to obtain dissociated DRG neurons. The cell suspension was centrifuged to remove the supernatant, and the cell pellet was re‐suspended in DMEM. The cells were then plated onto a 35‐mm culture dish containing pre‐coated coverslips.

Next, patch recordings were performed within 6 h after dissociation. Neurons were first visualized using a combination of epifluorescent illumination and differential interference contrast (DIC, 20‐40X) optics on an inverted microscope (Nikon TE2000). Under DIC, images of Dil‐positive neurons were displayed on a video monitor. The diameter of all neurons recorded was <40 µm. Neurons were patched in the whole‐cell configuration and recorded at a holding potential of −70 mV using a MultiClamp 700B amplifier. Seals (1–10 GΩ) between the glass electrode (2–5 MΩ resistance) and the cell were established in modified Tyrode's solution. After the whole‐cell configuration was established, the cell membrane capacitance and series resistance were electronically compensated.

Cells in the recording chamber were continuously bathed in Tyrode's solution. αβ‐me ATP stored in stock solution was diluted (10, 30, and 50 µM) in extracellular solution immediately before being used and it was held in a syringe connected to a silica column for delivery to the cells.^12^ The distance from the column mouth to the examined cell was ~100 µm. P2X currents were acquired using pClamp 9.0 software and the data were analyzed using Clampfit software. Neurons were considered P2X‐sensitive if αβ‐me ATP elicited an inward current with a peak amplitude >50 pA. For the patch‐clamp studies,^23^, ^24^ a minimum of 10 cells is required for each group. This provides at least 90% power. Therefore, we targeted 15 cells and the missing value was made by the failure to maintain the neuronal activities during the patch recording. The missing values were excluded during the analysis and the sample size for each group was shown in Figure 4.

### 2.6 Examination of the BP response

Based on our previous studies using the same approach,^12^, ^13^, ^25^ 12 rats in each group provide at least 90% power to detect a significant difference in MAP responses. Therefore, we targeted the sample size of 15 rats in each group for this experiment. The missing values were due to the death of the animal during this experiment and the missing values were excluded during the data analysis. The specific sample size of animals in each group is presented in Figure 5.

The rats were anesthetized with a mixture of 2%–5% isoflurane and oxygen and ventilated as described previously.^22^ The jugular vein and common carotid artery were cannulated. Fluids were delivered via the jugular vein while a pressure transducer was connected to the common carotid artery for measurement of the arterial BP. HR was calculated by the R–R interval in the electrocardiogram (ECG). To examine P2X‐mediated cardiovascular responses, a catheter (PE10) was inserted into the femoral artery for injection of αβ‐me ATP. In the PAD rats, a small incision was carefully made in the femoral artery distal to the previously occluded site. The catheter was then inserted into the artery toward the distal end to deliver the drug into the ischemic limb. During the experiments, baseline BP and fluid balance were maintained with a continuous infusion of saline and the body temperature was also maintained at ~37°C.

Decerebration was performed to eliminate the effects of anesthesia on the reflex pressor response. Prior to the procedure, dexamethasone (0.2 mg, i.v.) was injected to minimize brain stem edema. A transverse section was made anterior to the superior colliculus and extending ventrally to the mammillary bodies. The brain rostral to the section was then removed. This approach afforded the opportunity to examine the effect of the arterial injection of αβ‐me ATP on blood pressure without considering the confounding effects of anesthesia. After decerebration, anesthesia was withdrawn from the rats, and the animals were switched to a ventilator. Then experiments were performed 60 min later.

The reflex BP and HR responses to arterial injections of αβ‐me ATP were examined in WT, Occluded WT, KO, and Occluded KO rats. The concentrations of αβ‐me ATP were selected based on results of a previous study.^14^ The injection volume was ~0.1 ml according to the rat's body weight. Three concentrations of αβ‐me ATP (0.0625, 0.125, and 0.25 mM) were administrated in each rat and the order of drug administration was randomly assigned. The duration of the injection was 1 min, and an interval of 20 min was allowed between injections. At the end of the experiments, the animals were euthanized by inhalation of an overdose of isoflurane followed by cardiac puncture.

### 2.7 Statistical analysis

Unless specified, the data in this study are presented as the mean ± standard deviation (SD). The SPSS for Windows version 26.0 was utilized for all statistical analyses. Kolmogorov–Smirnov test was applied to evaluate the normality and Leven's test was applied to evaluate the equality of the variance. Once the data met the standard of normal distribution and equal variance, two‐way (genotype × ligation surgery) ANOVA was applied to compare the differences in BP and HR responses, the expression of P2X3 and the amplitude of P2X currents among the groups (WT, Occluded WT, KO, and Occluded KO). As appropriate, simple effect analysis was applied to compare the differences between specific groups. If the data did not meet the standard of normal distribution and equal variance, nonparametric Kruskal–Wallis test and Mann–Whitney test were applied. A *p* value less than 0.05 was considered as statistical significance.

## 3 RESULTS

### 3.1 Expression of ASIC3 and P2X3 in muscle DRG neurons

**FIGURE 2:**
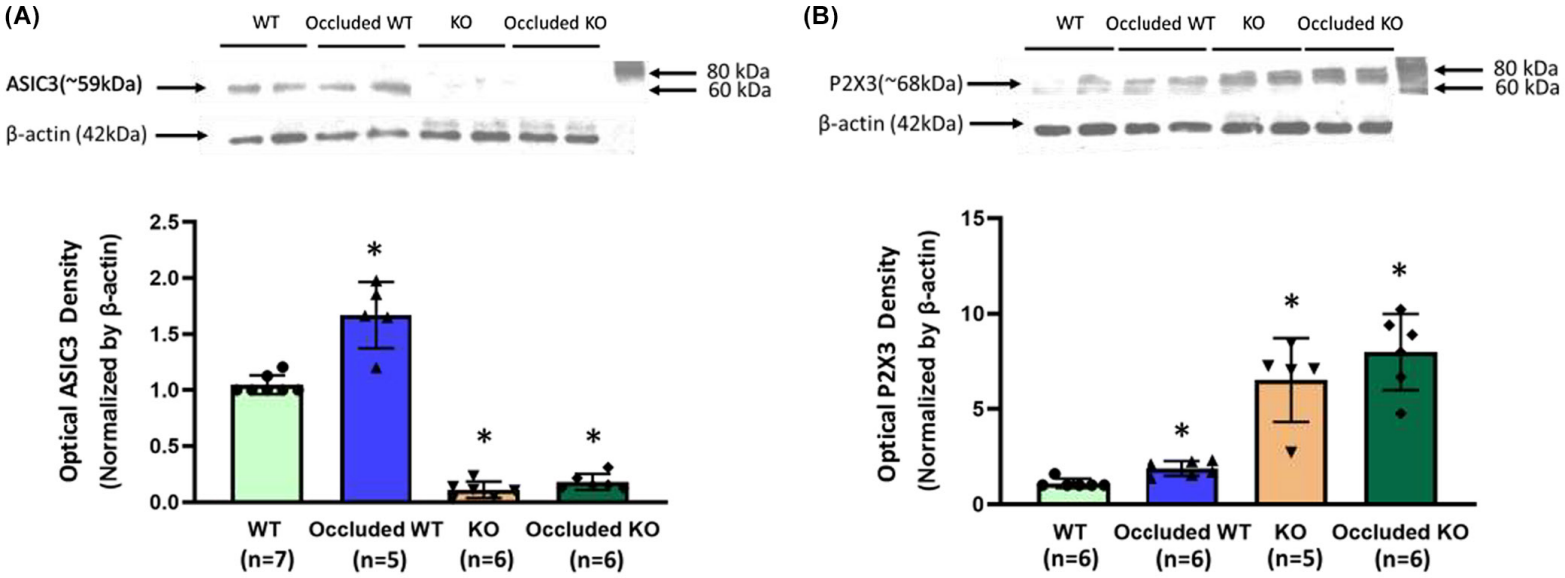
Protein levels of ASIC_3_ and P2X_3_ expression in DRG of WT, occluded WT, KO, and occluded KO rats. The optical density of ASIC_3_ and P2X_3_ receptor expression was normalized to that of the internal reference protein of β‐actin. (A): Two‐way ANOVA shown significant main effects on genotype (*F *= 392.50, *p *= 0.00, effect size partial Eta^2^ = 0.95), surgery (*F *= 31.83, *p *= 0.00, effect size partial Eta^2^ = 0.61) and interaction in genotype x surgery (*F *= 20.33, *p *= 0.00, effect size partial Eta^2^ = 0.50). Seventy‐two hours of femoral artery ligation increased the protein levels of ASIC_3_ expression in the L4‐L6 DRGs of WT rats (*n* = 5) compared with those of WT control rats (*n* = 7). ASIC_3_ expression was significantly decreased in KO rats without and with femoral artery occlusion (*n* = 6 in each group). *p < 0.05, vs. WT. (B) Kruskal–Wallis H test shown significant difference among four groups (*H *= 18.82, *p *= 0.00, effect size Eta^2^ = 0.83). Femoral artery occlusion increased the protein levels P2X_3_ expression in the L4‐L6 DRGs of WT rats compared with those of control rats (*n* = 6 in each group). Following ASIC_3_ KO, an increase in P2X_3_ protein expression was suppressed in occluded rats (*n* = 6) compared with KO rats not occluded (*n* = 5). Note that there was no significant difference in P2X3 receptor expression between KO and occluded KO rats. *p < 0.05, vs. WT

As shown in Figure 2A,B, in the western blot experiment, the protein expression of the ASIC3 and P2X3 receptor in the muscle DRG neurons of WT, occluded WT, KO, and occluded KO was examined. Compared with the WT, the ASIC3 expression was significantly lower in KO rats (optical density: 0.11 ± 0.07 in KO/*n* = 6 vs. 1.04 ± 0.08 in WT/*n* = 7; *p *= 0.00, effect size *Cohen's f *= 2.53) and higher in the occluded WT group (optical density: 1.67 ± 0.29 in occluded WT/*n* = 5 vs. 1.04 ± 0.08 in WT/*n* = 7; *p *= 0.00, effect size *Cohen's f *= 1.59). Unlike the effect of femoral artery occlusion on the WT rats, compared with the KO rats, ASIC3 expression was not significantly increased in occluded KO rats (optical density: 0.18 ± 0.07/*n* = 6; *p *= 0.43, effect size *Cohen's f *= 0.18 vs. KO rats).

In addition, the effect of ASIC_3_ KO on the protein levels of P2X3 in the DRGs was examined. Compared with that in WT, the density of the P2X3 signal was greater in the DRG tissues of occluded WT (optical density: 1.88 ± 0.38 in occluded WT vs. 1.09 ± 0.24 in WT control; Mann–Whitney *U* test shown *U *= 2.00, *p *= 0.01, effect size *r* = 0.767, *n* = 6 in each group). Compared with that in WT rats, P2X3 expression was significantly increased in KO rats (optical density: 6.51 ± 2.20; *n* = 5, Mann–Whitney *U* test shown *U *= 0.00, *p *= 0.00, effect size *r* = 0.87, vs. WT). However, unlike the effect of femoral occlusion on the WT rats, compared with the KO rats, the levels of P2X3 expression were not significantly increased in occluded KO rats (optical density: 7.99 ± 1.99/*n* = 6; Mann–Whitney *U* test shown *U *= 9.00, *p *= 0.27, effect size *r* = 0.33 vs. KO).

### 3.2 P2X currents in muscle DRG neurons

**FIGURE 3:**
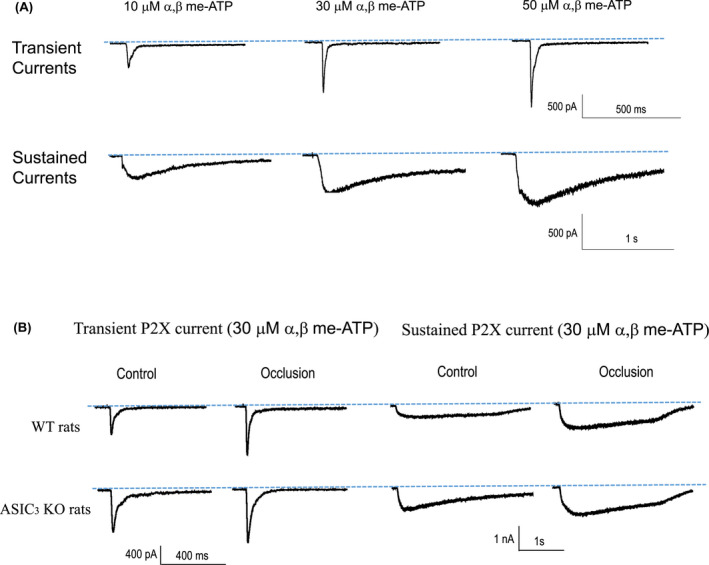
Representative P2X current traces induced by αβ me‐ATP in the four different groups. (A) Transient currents and sustained currents in muscle DRG neurons appeared to be a dose‐dependent manner after αβ me‐ATP. (B) The peak amplitude of transient and sustained P2X currents was amplified in the DRG neurons of occluded WT rats compared with WT controls. Following ASIC_3_ KO, the peak amplitude of transient and sustained currents became greater with application of αβ me‐ATP, but amplification of P2X currents was attenuated in occluded KO rats compared with KO rats

**FIGURE 4:**
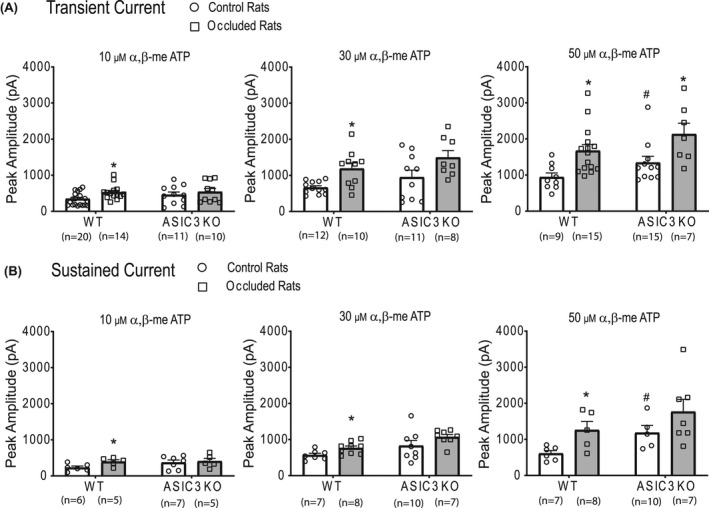
Averaged data of P2X currents in rat muscle DRG neurons in the four different groups. The average peak amplitude of transient P2X currents (A) and sustained currents (B) in the muscle neurons of WT rats and ASIC_3_ KO rats with or without femoral artery occlusion. 10, 30, and 50 µM αβ me‐ATP were applied onto muscle DRG neurons, respectively. The effects of αβ me‐ATP on the inward peak currents appeared in a dose‐dependent manner. For the transient current after the administration of αβ‐me ATP, Kruskal–Wallis *H* test shown no significant difference among four groups when the αβ‐me ATP concentration was 10 μM (*H *= 5.95, *p *= 0.11, effect size Eta^2^ = 0.05). Significant difference was found when the αβ‐me ATP concentration was 30 μM (Kruskal–Wallis *H* test shown *H *= 11.90, *p *= 0.01, effect size Eta^2^ = 0.25) and 50 μM (Kruskal–Wallis H test shown *H *= 14.70, *p *= 0.01, effect size Eta^2^ = 0.31). For the sustained current after the administration of αβ‐me ATP, no significant main effect for genotype and surgery, nor the interaction effect of genotype × surgery was found when the αβ‐me ATP concentration was10 μM (main effect of genotype: *F *= 1.47, *p *= 0.24, effect size partial Eta^2^ = 0.07; main effect of surgery: *F *= 2.50, *p *= 0.13, effect size partial Eta^2^ = 0.12; interaction effect of genotype × surgery: *F *= 1.07, *p *= 0.31, effect size partial Eta^2^ = 0.05). When the αβ‐me ATP concentration was 30 μM, significant difference was found (Kruskal–Wallis *H* test shown *H *= 11.70, *p *= 0.01, effect size Eta^2^ = 0.33). When the αβ‐me ATP concentration was 50 μM, significant main effect was found in both genotype (*F *= 4.41, *p *= 0.04, effect size partial Eta^2^ = 0.18) and surgery (*F *= 5.76, *p *= 0.03, effect size partial Eta^2^ = 0.23). No significant interaction effect of genotype x surgery was found (*F *= 0.02, *p *= 0.90, effect size partial Eta^2^ = 0.00). Increasing αβ‐me of ATP concentration led to the greater peak amplitude of P2X transient and sustained currents in DRG neurons of ASIC_3_ KO rats. # *p *< 0.05 between WT and ASIC_3_ KO. In WT rats, femoral artery occlusion increased peak amplitude of both transient and sustained P2X currents as αβ‐me ATP was applied, but the effect of femoral occlusion was not seen in KO rats when 10 and 30 µM of αβ me‐ATP were applied. *p < 0.05 between controls and femoral artery occlusion in both WT rats and ASIC_3_ KO rats. The number of DRG neurons recorded are shown in figures

As shown in Figure 3A,B, muscle DRG neurons from both WT rats and ASIC_3_ KO rats exhibited the typical transient and sustained current responses with activation of P2X receptors using αβ‐me ATP, and the inward peak currents appeared in a dose‐dependent manner. In specific, the transient current had a short rise time and a rapid inactivating rate (top, Figure 3A), while the sustained inward current gradually reached a peak and slowly inactivated (bottom, Figure 2A). In addition, typical traces show the greater amplitude of P2X currents of WT rats after femoral artery occlusion and femoral occlusion‐induced enhancement of P2X currents was inhibited in ASIC_3_ KO rats. Averaged data are presented in Figure 4. Specifically, in muscle DRG neurons of WT rats, the peak amplitudes of transient P2X currents evoked by 10, 30, and 50 μM of αβ‐me ATP was 347 ± 185 pA (*n *= 20), 663 ± 172 pA (*n *= 12), and 942 ± 350 pA (*n *= 9), respectively; in muscle DRG neurons of ASIC_3_ KO rats, they were 459 ± 256 pA (*n *= 11), 947 ± 628 pA (*n *= 10), and 1347 ± 579 pA (*n *= 11), respectively. Note that 10 and 30 of μM αβ‐me of ATP tended to increase the peak amplitude of P2X transient currents in DRG neurons of ASIC_3_ KO rats, whereas no significant difference was observed (10 μM: Mann–Whitney *U* test shown *U *= 84.00, *p *= 0.28, effect size *r* = 0.19, vs. WT; 30 μM: Mann–Whitney *U* test shown *U *= 52.00, *p *= 0.60, effect size *r* = 0.11, vs. WT). 50 μM of αβ‐me ATP increased the peak amplitude of P2X transient currents in DRG neurons to a greater degree in ASIC_3_ KO rats (1347 ± 579 pA, *n* = 15) than in WT rats (942 ± 350 pA, *n *= 9) (Mann–Whitney *U* test shown *U *= 23.00, *p *= 0.04, effect size *r *= 0.45). The similar results were also observed in the peak amplitudes of sustained P2X currents between WT rats and ASIC_3_ KO rats.

In this study, we further determined the effects of femoral artery occlusion on P2X current responses in muscle DRG neurons of ASIC_3_ KO rats and WT rats. As shown in Figure 3 in WT rats, femoral occlusion increased peak amplitude of both transient and sustained P2X currents as 10, 30, and 50 μM of αβ‐me ATP were applied. That is, the peak amplitude of transient P2X currents evoked by 30 μM of αβ‐me ATP was 663 ± 172 pA in non‐occluded (control) WT rats (*n *= 12) and 1189 ± 503 pA in occluded WT rats (*n *= 10) (Mann–Whitney *U* test shown *U *= 22.00, *p *= 0.01, effect size *r *= 0.53). The peak amplitude of the transient P2X currents evoked by 50 μM of αβ‐me ATP was increased from 942 ± 350 pA in control WT rats (*n *= 9) to 1674 ± 655 pA in occluded WT rats (*n *= 15) (Mann–Whitney *U* test shown *U *= 17.00, *p *= 0.00, effect size *r *= 0.61). However, no statistically significant differences in both transient and sustained P2X currents were found between control ASIC_3_ KO rats and occluded ASIC_3_ KO rats when αβ‐me ATP was applied except of the transient P2X current amplitude at 50 μM of αβ‐me ATP. With using 50 μM of αβ‐me ATP, the amplitude of the transient P2X currents in muscle DRG neurons was 1347 ± 580 pA in control ASIC_3_ KO rats (*n* = 11), and 2134 ± 796 pA in occluded ASIC_3_ KO rats (*n* = 7) (Mann–Whitney *U* test shown *U *= 14.00, *p *= 0.03, effect size *r *= 0.52).

### 3.3 Reflex BP response to P2X stimulation in muscle afferent nerves

**FIGURE 5:**
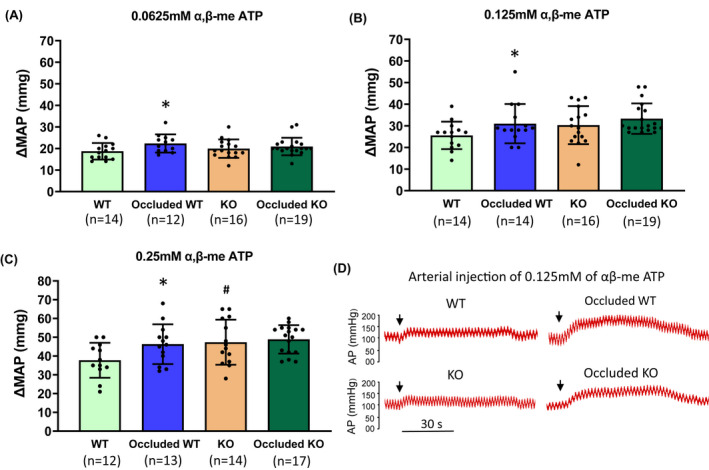
The pressor response evoked by arterial injection of αβ‐me ATP in the hindlimb circulation. (A–C) With arterial administration of αβ‐me ATP (0.0625, 0.125, and 0.25 mM), the enhancement of the MAP was greater in occluded WT rats than in WT control rats. *p < 0.05 vs. WT control rats. A significant increase of MAP induced by 0.25 mM of αβ‐me ATP was observed in ASIC_3_ KO rats. #*p *< 0.05 vs. WT rats. The same dosage of αβ‐me ATP failed to amplify MAP response in occluded ASIC_3_ KO rats as compared with KO controls. The number of animals are shown in figures. (D) Typical recordings of the changes in arterial pressure (AP) in the four different groups. Arterial injection of 0.125 mM of αβ‐me ATP increased arterial BP and this effect was enhanced in occluded WT rats; and enhancement of BP induced by femoral artery occlusion was attenuated in ASIC_3_ KO rats

In additional groups, we examined BP and HR responses to arterial injection of αβ‐me ATP (Table 1 and Figure 5). The basal MAP and HR prior to arterial administration of 0.0625mM (basal MAP: *p *= 0.01, effect size *Cohen's f *= 0.342; basal HR: *p *= 0.02, effect size *Cohen's f *= 0.294) and 0.25 mM (basal MAP: *p *= 0.03, effect size *Cohen's f *= 0.314; basal HR: *p *= 0.03, effect size *Cohen's f *= 0.286) αβ‐me ATP were observed to be lower in KO rats than in WT rats. Meanwhile, for the basal HR prior to arterial administration of 0.125 mM αβ‐me ATP, a significant main effect was found in surgery (*F *= 3.03, *p *= 0.00, effect size partial Eta^2^ = 0.516). And the main effect of genotype was found marginally significant (*F *= 3.03, *p *= 0.08, effect size partial Eta^2^ = 0.04). No significant main effect of surgery was found (*F *= 2.42, *p *= 0.12, effect size partial Eta^2 ^= 0.04). Of note, the basal HR was appeared to be lower in KO rats than in WT rats (*p *= 0.02, effect size *Cohen's f *= 0.28) (Table 1). As shown in Figure 5A–C, following αβ‐me ATP injection, the MAP response in occluded WT rats was significantly higher than that in WT rats (0.0625 mM: 22 ± 4 mmHg in occluded WT rats/*n* = 12 vs. 18 ± 4 mmHg in WT rats/*n* = 14, *p *= 0.01, effect size *Cohen's f *= 0.35; 0.125 mM: 31 ± 9 mmHg in occluded WT rats/*n* = 14 vs. 25 ± 6 mmHg in WT rats/*n* = 14, Mann–Whitney *U* test shown *U *= 55.00, *p *= 0.047, effect size *r *= 0.38; 0.25 mM: 46 ± 10 mmHg in occluded WT rats/*n* = 13 vs. 37 ± 9 mmHg in WT rats/*n* = 12, *p *= 0.04, effect size *Cohen's f *= 0.30). It is noted that there were no statistically significant differences in the MAP response to αβ‐me ATP injection between KO rats and occluded KO rats for three dosages of αβ‐me ATP injection. Compared with that in WT rats, the enhancement in the MAP response induced by 0.25 mM αβ‐me ATP injection was higher in KO rats (47 ± 12 mmHg/*n* = 14; *p *= 0.017, effect size *Cohen's f *= 0.34 vs. WT rats). Apart from the significant difference in HR response between WT and KO rats after the injection of 0.0625 mM αβ‐me ATP (10 ± 3 bpm in WT rats/*n* = 10 vs. 7 ± 3 mmHg in KO rats/*n* = 14, *p *= 0.03, effect size *Cohen's f *= 0.30), there was no significant difference in the HR response observed among groups after the injection of 0.125 (*p *= 0.64, effect size *Cohen's f *= 0.12) and 0.25 mM of αβ‐me ATP (*p *= 0.15, effect size *Cohen's f *= 0.07). Figure 5D further shows typical recordings of the changes in BP after arterial injection of 0.125 mM of αβ‐me ATP in the four different experimental groups.

**TABLE 1:** The baseline values for blood pressure (BP) and heart rate (HR) in 0.0625, 0.125, and 0.25 mM αβ‐me ATP administration experiments

	0.0625 mM				0.125 mM				0.25 mM			
	WT rats	Occluded rats	KO rats	Occluded KO rats	WT rats	Occluded rats	KO rats	Occluded KO rats	WT rats	Occluded rats	KO rats	Occluded KO rats
Sample size (*N*)	13	12	16	19	13	14	16	19	12	13	14	17
MAP (mmHg)	106 ± 10	101 ± 14	92 ± 12*	99 ± 14	105 ± 12	97 ± 16	93 ± 10	96 ± 15	111 ± 17	98 ± 13	90 ± 11*	98 ± 16
Sample size (N)	12	22	11	25	12	22	12	25	7	22	11	25
Heart rate (bpm)	455 ± 57	498 ± 40	356 ± 34*	385 ± 59	455 ± 43	496 ± 40	369 ± 42*	371 ± 59	439 ± 37	486 ± 37	374 ± 44*	368 ± 62

Note

BP, blood pressure; KO, ASIC3 knockout rats. The basal MAP and HR before injection of 0.0625 and 0.25 mM αβ‐me ATP were significantly different between WT and KO rats; The basal HR before injection of 0.125 mM αβ‐me ATP was significantly different between WT and KO rats (all *p *< 0.05). All the data were present as mean ± standard deviation (SD).

## 4 DISCUSSION

In the present study, we first observed in the ASIC3 KO rats: (1) upregulated P2X3 expression in muscle DRG neurons; (2) increased transient and sustained P2X currents in muscle DRG neurons; and (3) amplified MAP response to αβ‐me ATP injected into the hindlimb circulation. We subsequently found that (1) femoral artery occlusion enhanced P2X3 expression and amplified the activity of P2X currents in the WT animals, but not in the ASIC_3_ KO rats; and (2) femoral artery occlusion exaggerated MAP response induced by αβ‐me ATP in the WT animals, but not in the KO group.

With a tremendous amount of affected population, PAD induces a great healthcare burden for the society.^26^, ^27^ In spite of the intensive focus on the autonomic responses during exercise activity in cardiovascular diseases and intervention strategies to improve the vascular function, few attentions have been paid on the involvement of signal pathways and molecular mediators responsible for the exaggerated exercise pressor reflex in PAD. Considering that the mechanisms about the ASIC3 and P2X3 studied in the human PAD patients are currently lacking, the investigations on these receptors in regulating the exercise pressor reflex in an animal model, as well as the interaction among the molecular components are anticipated to provide basic evidence for the pharmacological intervention used for the clinical treatment and management of PAD.

Adenosine triphosphate (ATP), lactate, and protons are among the chief metabolites produced within the muscular fibers during the exercise. ATP released into the muscle interstitium stimulates P2XRs, the P2 purinoceptors receptors family for conducting ATP signals, in the muscle afferent nerves. The primary thin fiber Group III and IV muscle afferent nerves are responsible for the increasing BP induced by arterial injection of αβ‐me ATP into the hindlimb circulation via P2X3 and P2X2/P2X3.^12^ In addition, the process of ATP hydrolysis induces the production of protons.^28^ In combination of the lactate, one of the chief products of the anaerobic metabolism, the pH value reduces and the acid sensing ion channels (ASICs) are stimulated. A total amount of six different proteins (ASIC1a, 1b, 2a, 2b, 3 and 4) consist of the ASICs family. In particular, the ASIC3 is predominantly located in the primary DRG neurons and activated as pH drops less than ~7. In exercising and/or ischemic muscles, the pH value of the muscle interstitium decreases to 6.7–7.0 and it could be even lower to 6.4 following the maximal exercise.^29^, ^30^ As a result, activation of the ASIC3 in muscle afferent nerves contributes to the augmented sympathetic nerve and cardiovascular activities in response to the exercise and/or muscle ischemia condition. Considering the close relationship among those metabolites in exercising muscles, we therefore have focused on the interaction of the P2X3 and the ASIC3 in muscle afferent nerves. The technique of in vivo ASIC3 knockdown in Wistar Kyoto rats provides us with an opportunity to determine the role played by ASIC_3_ in regulating the activities of P2X signaling pathways with respect to the exaggerated exercise pressor reflex in PAD.

In our present study, we first identified the expression and functions P2X_3_ in muscle afferent neurons by using multiple approaches. That is, western blot analysis was employed to evaluate the protein levels of P2X3 receptor expression in DRGs; and patch‐clamp technique was used to examine current response in muscle DRG neurons with activation of P2XRs. We then determined the BP response induced by stimulation of P2X3 in muscle afferent nerves following ASIC3 KO in animals whose hindlimbs were freely perfused and underwent for 3 days of femoral artery occlusion.

Interestingly, it was found that there was an increasing P2X3 expression in the muscle DRG neurons of the ASIC_3_ KO rats. Consistently, it has also been revealed that the ASIC3 and P2X3 were not only co‐expressed in the rat DRG neurons, but also that the activation of ASIC3 played an inhibitory effect on the activity of P2X3 when the P2X currents were assessed in cell lines and in DRG neurons.^15^ In contrast, the application of αβ‐me ATP was not observed to affect the ASIC3 currents in DRG neurons.^15^ This previous study suggested the inhibitory effect between ASIC3 and P2X3 is unidirectional from ASIC3 to P2X3. In another study, αβ‐me ATP was injected into the femoral artery to activate the P2X3 in muscle afferent nerves under different interstitial pH conditions of 7.4, 6.5, and 5.5.^31^ The results of this previous study revealed that BP was increased following the arterial injection of αβ‐me ATP under all pH values. However, compared with pH 7.4, the BP response by αβ‐me ATP was significantly lower with less pH values. Also, the lowered αβ‐me ATP‐induced BP response was largely reversed by a blockade of ASICs using amiloride, indicating that there is a functional linkage between P2X3 and ASICs. In our present study, we have also demonstrated that the amplitude of P2X currents in muscle DRG neurons became gradually greater with increasing concentrations of αβ‐me ATP following ASIC3 KO. It is therefore well reasoned for the greater BP response to a higher dosage of αβ‐me ATP in ASIC3 KO rats observed in the present study. Taken together, it is likely that the amplified BP response to αβ‐me ATP resulted from the ASIC3 KO was due to the increasing expression and activities of P2X3 signaling pathways in the ASIC3 KO rats.

Another interesting finding deserves our attention is that, in western blot experiment, the P2X3 protein expression was markedly (>6 folds) increased in ASIC3 KO rats but the effect of ASIC3 KO on current and BP responses to ATP was modest (~2 folds) and only was observed at higher concentrations of ATP. The similar result was also noticed in the BP responses in ASIC3 KO rats. To interpret these findings, it should be noted that αβ‐me ATP is an agonist affecting both P2X2/P2X3 complex and P2X3 receptors. The functional alternation of the BP response and the P2X currents is therefore a combination of the stimulation of P2X2/P2X3 and P2X3. Considering the focus scope of the present study, in the western blot we only detected the expression of P2X3 expression. It is presumed that the less increment in BP and P2X currents versus the protein expression could be due to the engagement of P2X2/P2X3 complex. Meanwhile, it should be also recognized that the P2X3 is synthesized in the cytoplasm and transferred to the cell membrane for further functioning. It is therefore speculated that the increasing amount of P2X3 protein is mostly stored in cytoplasm instead of the membrane. A total level of P2X3 seen using the current western blot analysis is unlikely reflective of its functions leading to changes of P2X currents and BP response. Notwithstanding the above mentioned, whether the ASIC3 KO could affect the quantity or function of the P2X2/P2X3, and the capability of P2X3 being transferred into the cell membrane is still unknown and further studies are needed to elucidate this potential mechanism.

In terms of the BP response to activation of P2X receptors, the findings in the WT rats and ASIC3 KO rats in the present study are somehow consistent with a previous study which examined the exercise pressor reflex in the ASIC3 KO rats.^20^ The result suggests that ASIC3 did not result in a decrease in pressor response to static muscle contraction by its genetic knockout per se. In this previous study it was proposed that components other than ASIC3 play a role in regulating the BP response during muscle contraction.^20^ Based on our results, ASIC3 may play an inhibitory role in modulating both protein expression and functions of the P2X3 in the muscle afferent nerves. With ASIC3 KO, de‐inhibition on the activities of P2X3 signaling pathways leads to amplification of the P2X current and BP responses with the receptor stimulation.

Another interesting finding of the present study was that femoral artery occlusion did not enhance the P2X3 protein expression, P2X current and BP responses in the ASIC3 KO rats as it did in WT rats. For the P2X currents, an exception was that transient current in response to the highest concentration of αβ‐me ATP (50 µM) was increased in occluded ASIC3 KO rats. It is noted that, in a previous study^19^ using ASIC3 KO rats, the pressor response to muscle contraction was examined following femoral artery occlusion, showing that the exercise pressor reflex was attenuated in the occluded ASIC3 KO rats. It is speculated that there is a ceiling effect such that the effect of the femoral occlusion in KO cannot be observed. In a general agreement, in our present study, the ASIC3 protein in KO rats was not increased by the femoral artery occlusion. This suggests the CRISPR‐induced ASIC3 KO prevented the gene transcription and protein functioning under the ischemic condition linking to the role of P2X3.

A number of studies have shown that the ASIC_3_ and P2X_3_ are co‐expressed in the DRG neurons. The addition of the nerve growth factor, which is elevated under the ischemia condition, in the cell culture medium induced the co‐increases in ASIC3 and P2X3 in the DRG neurons.^15^ It is also reported that those two receptors formed a protein complex as a cognate receptor to regulate the neuronal activity. During ischemia, cells are swollen and this change enhances the permeability of ATP through the cell membrane into the interstitial space.^32^ A previous study also suggests that the ASIC3 is sensitized by forming a molecular complex with P2X5 when it is binding with ATP increased and accumulated in the ischemic tissues.^33^ This conclusion was supported by the co‐expression of P2X5 and ASIC3 in the DRG neurons, and the evidence that the ASIC current is sensitized by ATP. With the genetic deletion of ASIC3, the P2X5 fails to bind with the ASIC3 and its subsequent potentiating activity on the sensory neurons is reduced. With the decreased ASIC3 activation‐induced neuronal activity, there is a less stimulation of the cell membrane and therefore the ATP released into the interstitial space is decreased. As a result, the P2X_3_ expression in the DRG neurons failed to significantly increase in the occluded ASIC3 KO rats in the present study. It is also speculated that, although the ASIC3 plays a role in inhibiting the expression and function of P2X3, the ischemia‐induced expression of P2X3 may require the co‐existence of the ASIC3 genes/proteins. However, whether those two receptors are associated at the genetic level is still unknown and need to be confirmed by additional studies.

Baseline BP in WT rats and KO rats: Regarding cardiovascular responses to activation of muscle afferents’ P2X3, it is interesting to observe a decreased baseline of BP and HR in ASIC3 KO rats. This is partly consistent with the previous study from Cheng et al.,^34^ in which the ASIC3 −/− mice demonstrated a blunt BP response under stress condition and the ratio of the low frequency power spectral density was lower in ASIC3 −/− mice than the ASIC3 +/+ mice. This indicates a blunted effect on the sympathetic control of cardiovascular activity following the global abolishing of the ASIC3. Meanwhile, it was also indicated in another study^35^ that, compared with other subtypes of ASICs such as ASIC2, the ASIC3 is more vital in sympathetic regulation by distributing in the muscle DRG neurons. Therefore, it is not surprised that the basal BP and HR were lower in KO rats than WT rats in the present study. Moreover, we performed BP and HR measurement in the decerebrated rats to examine the specific impact of ASIC3 KO on the peripheral neural regulation of the cardiovascular activity. This approach eliminates the effect of ‘central command’ on basal BP and HR, therefore distinguished the present study and previous one^34^ with animals in conscious status.

Nonetheless, in the current study, we observed a lower resting BP in KO rats. This result may affect BP response induced by P2X activation using α, β‐me‐ATP. For example, a BP response to stimulation of muscle afferent is greater as resting BP is lower due to an engagement of arterial baroreflex.^36^ Although there was no significant difference in resting BP between WT rats and KO with and without femoral artery occlusion, noticeable difference was seen. We cannot rule out that the different BP response after occlusion in WT rats and KO rats was due to resting BP. Additional studies will be interesting to determine the role played by arterial baroreflex in regulating BP response to activation of P2X receptor in muscle afferent nerves in KO rats.

In conclusion, our study provides novel data about the protein expression and function of P2X3 receptors in muscle sensory afferent after the genetic deletion of ASIC3. Compared with WT rats, augmented P2X3‐mediated pressor response is blunted by ASIC3 KO in PAD rats and this is accompanied with downregulated P2X3 expression and suppressed activities in P2X3 signaling pathways in muscle afferent neurons.

## CONFLICT OF INTEREST

The authors declare no conflict of interest.

## AUTHOR CONTRIBUTIONS

Lu Qin designed, performed the research, analyzed data, and wrote the paper; Qin Li performed research and analyzed data; Jianhua Li designed the research, analyzed data, and wrote the paper. All authors reviewed the results and approved the final version of the manuscript.
